# COVID-19 vaccine hesitancy and attitudes in Pakistan: a cross-sectional phone survey of major urban cities

**DOI:** 10.1186/s12889-023-15905-3

**Published:** 2023-06-09

**Authors:** Adnan Ahmad Khan, Mujahid Abdullah, Razia Aliani, Amal Fatima Mohiuddin, Faisal Sultan

**Affiliations:** 1 Research and Development Solutions, Islamabad, Pakistan; 2 Ministry of National Health Services, Regulation and Coordination, Islamabad, Pakistan; 3 Akhter Hameed Khan Foundation, Islamabad, Pakistan; 4 Shaukat Khanum Memorial Cancer Hospital and Research Centre, Lahore, Pakistan

**Keywords:** COVID-19, Vaccine willingness, Vaccine hesitancy, Phone survey, Pakistan

## Abstract

**Background:**

COVID-19 mass vaccination is the only hopeful savior to curb the pandemic. Vaccine distribution to achieve herd immunity is hindered by hesitance and negative attitude of the public against COVID-19 vaccination. This study aims to evaluate the vaccine hesitancy and attitudes in major cities in Pakistan as well as their determinants.

**Methods:**

A cross-sectional telephonic survey was conducted in June 2021 in major cities of Pakistan including Karachi, Lahore, Islamabad, Peshawar, and Gilgit, from unvaccinated urban population aged 18 years or older. Random Digit Dialing through multi-stage stratified random sampling was used to ensure representation of each target city and socio-economic classes. Questionnaire collected information on socio-demographics, COVID-19-related experiences, risk perception of infection, and receptivity of COVID-19 vaccination. Multivariate logistic regression analyses were performed to identify key determinants of vaccine hesitancy and acceptance.

**Results:**

The prevalence of vaccinated population in this survey was 15%. Of the 2270 respondents, 65% respondents were willing to vaccinate, while only 19% were registered for vaccination. Factors significantly associated with vaccine willingness were older age (aOR: 6.48, 95% CI: 1.94–21.58), tertiary education (aOR: 2.02, 95% CI: 1.36, 3.01), being employed (aOR: 1.34, 95% CI: 1.01, 1.78), perceived risk of COVID-19 (aOR: 4.38, 95% CI: 2.70, 7.12), and higher compliance with standard operating procedures (aOR: 1.72, 95% CI: 1.26, 2.35). The most common vaccine hesitancy reasons were ‘no need’ (*n* = 284, 36%) and concerns with ‘vaccine safety and side effects’ (*n* = 251, 31%), while most reported vaccine motivation reasons were ‘health safety’ (*n* = 1029, 70%) and ‘to end the pandemic’ (*n* = 357, 24%).

**Conclusions:**

Although our study found 35% hesitancy rate of COVID-19 vaccine, there were noticeable demographic differences that suggest tailored communication strategy to address concerns held by most hesitant subpopulation. Use of mobile vaccination facilities particularly for less mobile and disadvantaged, and implementation and evaluation of social mobilization strategy should be considered to increase overall COVID-19 vaccination acceptance and coverage.

**Supplementary Information:**

The online version contains supplementary material available at 10.1186/s12889-023-15905-3.

## Introduction

COVID-19 emerged as one of the biggest health and humanitarian crises in recent times and persists with resurgent waves [1]. As of February 23, 2022, more than 424 million cases and over 5.89 million deaths from COVID-19 have been reported worldwide, while Pakistan has reported 1.5 million cases and 0.03 million deaths [2]. Until recently the mainstay of epidemic control was to limit contacts between individuals, either by the use of personalized barriers such as masks of protective gear or by limiting contacts among people through lockdowns [3]. These were attended by considerable social and economic costs, and many countries still saw considerable transmission, hospitalizations, and deaths [3].

The availability of COVID-19 vaccines from late 2020 onwards opened up the possibility of curbing the epidemic by rapidly inducing immunity in societies through mass vaccinations. Researchers have estimated that, depending on the efficacy of the vaccine being used, 60–80% of a population needs to be vaccinated to control transmission [4, 5]. Conversely, a lag in vaccination can lead to emergence and transmission of more contagious and severe variants, some of which may possibly override immunity conferred by vaccines or previous infection [6, 7].

However, the expedited development and approval process for new vaccines has fueled doubts about their safety and efficacy [8]. The fact that this is the largest mass vaccination effort in history means that nearly all humans may be affected, including many that may otherwise have resisted vaccinations or other health interventions. Some of these concerns play across political affiliations, which have exacerbated vaccine hesitancy [9].

Populations residing in low- and middle-income countries have expressed considerable vaccine hesitancy of 38% due to mistrust and fears of potential side effects [10]. Such fears may resonate in Pakistan, where previous vaccine hesitancy about polio, often grounded in myths, misconception, or misinformation [11], has translated into low childhood vaccination rates for vaccine-preventable diseases [12]. Effective vaccine roll-out requires a comprehensive understanding of such vaccine hesitancy and determinants of people’s motivation to receive the vaccine.

This study was conducted in June 2021, when Pakistan’s vaccination effort was in its 3^rd^ month. Since COVID-19 is an epidemic of proximity that has mainly affected populous cities [13], our study was conducted in the 5 densest urban centers of Pakistan. To our knowledge, there were no studies documenting vaccine hesitancy among the general public in Pakistan during this period. Although a few studies were published later in 2021, these either covered healthcare workers or collected data through convenience sampling which was not representative of major cities of Pakistan [14–18].

This study explored the intent of individuals in Pakistani cities to receive COVID-19 vaccination and assess factors associated with acceptance or refusal of the COVID-19 vaccine, in order to better inform the ongoing vaccination campaign. Given constraints of collecting data during an upsurge in viral transmission at the time of study design (but not necessarily during data collection), data were collected randomly through telephonic interviews.

## Methods

### Study design

A cross-sectional survey was conducted via telephone during June 2021 in five densest urban centers of Pakistan: Karachi, Lahore, Islamabad, Peshawar, and Gilgit. All unvaccinated urban individuals that were aware of COVID-19 and its vaccination drive in Pakistan, residing in these five cities, and aged 18 years or older were eligible to participate. Those who refused to participate in the study or did not consent were automatically excluded from the study. Those who were already vaccinated, unaware of COVID-19 or unaware of Pakistan’s vaccination program, were also excluded from the study.

### Sampling

**Fig. 1:**
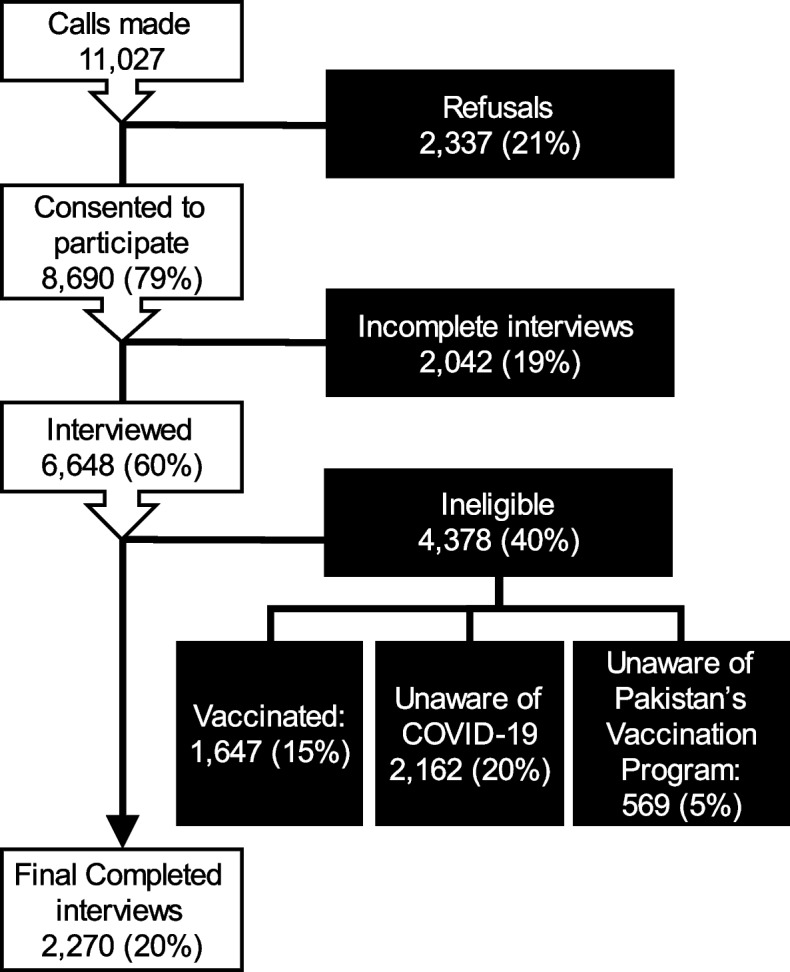
Study eligibility flow chart

Our final sample comprised of 2270 individuals from Karachi (750), Lahore (650), Islamabad (385), Peshawar (385), and Gilgit (100) (Fig. 1). Sample size was calculated individually for each city to allow a confidence interval of 95% and less than 5% margin of error and then adjusted for city population and wealth status of respondents. Respondents were reached from among the 70 million plus customers of the telecom services provider Jazz, using Random Digit Dialing (RDD) through an automatic dialer that randomly dialed numbers and connected calls to agents upon successful dial tone. Interviews were conducted by a full-time research team from Jazz that routinely conducts market surveys and was further trained by the investigators. Respondents were stratified into tiers in terms of average revenue per user (ARPU) data from Jazz as a surrogate for wealth status. Post-stratification weights were applied to represent sampling frame of each city.

### Study tools

The primary study outcome was intent to receive vaccination, defined as the percentage of respondents who answered, ‘Yes’ to the question, ‘Would you be willing to take COVID-19 vaccine offered by the government?’. The survey tool included questions on socio-demographic characteristics of the participants, along with questions about the level of COVID-19 awareness, perceptions about the disease, self or family experience with COVID-19, level of Standard Operating Procedures (SOPs) compliance, sources of COVID-19 information (e.g., newspapers, TV, radio, etc.), knowledge/awareness of the available vaccines, and willingness to accept COVID-19 vaccination. The complete questionnaire is provided in Supplementary Material (Table S1). Prior to implementation, the survey questionnaire was pilot-tested among 25 respondents (15 females and 10 males) with assumed similar characteristics to our sample. The questionnaire was improved for internal validity where issues arose.

### Statistical analysis

Sociodemographic characteristics of respondents were expressed as frequencies and percentages. Reasons for vaccine willingness and hesitancy were also reported descriptively. Odds ratios adjusted for sex, age group, education level, and occupation were calculated for socioeconomic status, sources of information, risk perception of COVID-19, prior self- or family experience with COVID-19 and SOP compliance score. Determinants of vaccine acceptance and registration to receive vaccinations by respondents were modelled using multivariate logistic regression analysis since the dependent variables were binary in nature. We estimated two multivariate logistic regression models with heteroskedasticity-adjusted robust standard errors:

$$logit=log(\frac{P}{1-P})={\beta }_{0}+\sum_{i=1}^{n}{\beta }_{i}{{\varvec{X}}}_{{\varvec{i}}}+ {\varepsilon }_{i}$$

where *P* (defined as the probability of success) took a value of 1 if an individual responded ‘Yes’ to the question ‘Would you be willing to take COVID-19 vaccine offered by the government?’ and 0 otherwise for model 1. Furthermore, in model 2, *P* took a value of 1 if the answer to ‘Have you registered for COVID-19 vaccination?’ was a ‘Yes’ and 0 otherwise. The independent variables were same for both models which included socio-demographic characteristics (city, sex, age group, education level, and employment status), risk perception of COVID-19, prior self- or family infection of COVID-19, and SOPs compliance score.

Post-stratification weights were computed in all regression analyses to match the distribution of population among cities with the target proportions from most recent census data. Stata 17 software was used for statistical analysis.

## Results

### Socio-demographic characteristics of the respondents

A total of 11,027 calls were attempted; 21% (*n* = 2,337) declined to participate in the study and 19% (*n* = 2,042) did not complete the survey. Of the remaining 6,648 respondents, 4,378 did not meet the inclusion criteria (Fig. 1) including 24% that had received at least one dose of vaccine. A total of 2,270 (20% of total calls) interviews were completed.

The mean age of the respondents was 30 years, with a female to male ratio of 1:3, with slightly lower proportion of female respondents from Gilgit and Peshawar. Most respondents were educated up to secondary level (Grade 8, 44%) or higher (17%); only 16% (*n* = 366) were either uneducated or educated less than the primary (Grade 5) level. Majority of the respondents were working (67%), and 45% were from lower or lower-middle socioeconomic strata (Table 1).

**Table 1:** Socio-demographic characteristics of participants

	**Total ** ***n*** ** = 2270**	**Karachi ** ***n*** ** = 750**	**Lahore ** ***n*** ** = 650**	**Islamabad ** ***n*** ** = 385**	**Peshawar** ***n*** ** = 385**	**Gilgit ** ***n*** ** = 100**
**Distribution of sample**		33%	29%	17%	17%	4%
**Sociodemographic characteristics**						
** Sex**						
Male	1748 (77)	567 (76)	434 (67)	303 (79)	351 (91)	93 (93)
**Age group**						
18—30	1334 (59)	451 (60)	399 (61)	226 (59)	209 (54)	49 (49)
31—40	508 (22)	154 (20)	138 (21)	86 (22)	96 (25)	34 (34)
41—50	231 (10)	69 (9)	65 (10)	45 (12)	42 (11)	10 (10)
51—60	89 (4)	31 (4)	28 (4)	11 (3)	15 (4)	4 (4)
Above 60	38 (2)	11 (2)	12 (2)	8 (2)	4 (1)	3 (3)
**Educational level**						
None or less than primary	366 (16)	151 (20)	105 (16)	46 (12)	54 (14)	10 (10)
Primary to middle (< grade 9)	432 (19)	147 (20)	137 (21)	68 (18)	65 (17)	15 (15)
Secondary to intermediate	1006 (44)	318 (42)	299 (46)	164 (43)	170 (44)	55 (55)
Tertiary (Bachelor or higher)	396 (17)	99 (13)	103 (16)	98 (26)	77 (20)	19 (19)
**Occupation**						
** Not Working**						
Unemployed	104 (5)	38 (5)	24 (4)	16 (4)	20 (5)	6 (6)
Student	235 (10)	65 (9)	79 (12)	36 (9)	51 (13)	4 (4)
Retired	15 (1)	2 (0.3)	4 (0.6)	6 (1.6)	3 (0.8)	0 (0)
Housewife	317 (14)	117 (16)	136 (21)	43 (11)	15 (4)	6 (6)
**Self-employed**						
Small business	576 (25)	171 (23)	130 (20)	108 (28)	132 (34)	35 (35)
Large businessmen	17 (1)	10 (1.3)	2 (0.3)	3 (0.8)	2 (0.5)	0 (0)
**Salaried**						
Unskilled worker	301 (13)	124 (16)	83 (13)	43 (11)	41 (11)	10 (10)
Low skilled worker	498 (22)	163 (22)	155 (24)	91 (24)	78 (20)	11 (11)
Skilled worker	123 (5)	21 (3)	29 (4)	27 (7)	21 (6)	25 (25)
**Socioeconomic status**						
High	339 (15)	89 (12)	73 (11)	69 (18)	85 (22)	23 (23)
Upper Middle	309 (14)	101 (14)	75 (12)	63 (16)	59 (15)	11 (11)
Middle	516 (23)	152 (20)	174 (27)	88 (23)	74 (19)	28 (28)
Lower Middle	426 (19)	138 (18)	129 (20)	73 (19)	67 (17)	19 (19)
Low	591 (26)	228 (30)	191 (29)	79 (20)	78 (20)	15 (15)

Estimates are presented as frequency (%). Where % do not add up to 100, all participants did not respond to that question. Where % exceeds 100, the questions were multi-select

Television (55%) and social media (37%) were the leading sources of information about COVID-19, related preventive measures, and vaccines. Information from workplace or educational institutions, radios, and religious leaders was mentioned by < 1% respondents. This pattern was similar across all cities.

Lived experience of COVID-19 infection was uncommon. Only 3.5% of respondents reported having experienced the infection themselves and 5% reported it in a family member. Only 1% reported hospitalization for themselves or a family member. Experience of COVID-19 infection was more frequent in Islamabad (6% for self and 11% for a family member) and Peshawar (7% and 9% respectively).

Despite this, most (80%) respondents believed COVID-19 is an extreme risk to the community, while only 5% (*n* = 109) believed COVID-19 poses no risk. Most respondents reported they were either moderately (31%) or highly compliant with government recommended SOPs for public gatherings (55%) (Table 2).

**Table 2:** Sources of COVID-19 information, prior experience and risk perception

	**Total (** ***n*** ** = 2270)**	**Karachi (** ***n*** ** = 750)**	**Lahore (** ***n*** ** = 650)**	**Islamabad (** ***n*** ** = 385)**	**Peshawar (** ***n*** ** = 385)**	**Gilgit (** ***n*** ** = 100)**
**Source of COVID-19 information**						
TV	1251 (55)	401 (53)	396 (61)	196 (51)	198 (51)	60 (60)
Internet/social media	795 (35)	244 (32)	184 (28)	153 (40)	171 (28)	43 (43)
Word of mouth/ Family/friends	531 (23)	189 (25)	147 (23)	81 (21)	99 (26)	15 (15)
Health provider	227 (10)	74 (10)	57 (9)	41 (11)	42 (11)	13 (13)
Call/SMS	145 (6)	51 (7)	23 (3)	34 (9)	27 (7)	10 (10)
Newspaper	107 (5)	26 (3)	11 (2)	24 (6)	37 (10)	9 (9)
Government institutes	76 (3.3)	26 (3.5)	13 (2)	15 (3.9)	9 (2.3)	13 (13)
Workplace/ Educational institutes	20 (0.9)	7 (0.9)	8 (1.2)	1 (0.3)	2 (0.5)	2 (2)
Radio	8 (0.4)	4 (0.5)	0 (0)	1 (0.3)	3 (0.8)	0 (0)
Religious leaders	5 (0.2)	3 (0.4)	0 (0)	1 (0.3)	1 (0.3)	0 (0)
None	270 (12)	104 (14)	72 (11)	54 (14)	32 (8)	8 (8)
**Prior experience of COVID-19**						
By the respondent	81 (4)	11 (2)	16 (2)	24 (6)	27 (7)	3 (3)
By a family member	114 (5)	15 (2)	19 (3)	42 (11)	33 (9)	5 (5)
**Hospitalization due to COVID-19**	26 (1.2)	1 (0.1)	3 (0.5)	9 (2.3)	12 (3.1)	1 (1)
**COVID-19 risk perception**						
Yes, extremely risky	1811 (80)	572 (76)	544 (84)	295 (77)	322 (84)	78 (78)
Moderately risky	193 (8)	66 (9)	44 (7)	42 (11)	27 (7)	14 (14)
Not risky at all	109 (5)	47 (6)	25 (4)	22 (6)	10 (3)	5 (5)
**SOP compliance score**						
Low compliance	311 (14)	105 (14)	68 (10)	64 (17)	57 (15)	17 (17)
Moderate compliance	695 (31)	231 (31)	180 (28)	113 (29)	132 (35)	39 (39)
High compliance	1255 (55)	414 (55)	397 (62)	207 (54)	193 (50)	44 (44)

Estimates are presented as frequency (%), with the denominator being all those who completed the survey. Where % do not add up to 100, all participants did not respond to that question. Where % exceeds 100, the questions were multi-select

### Intention to vaccinate and registration to receive vaccination

**Fig. 2:**
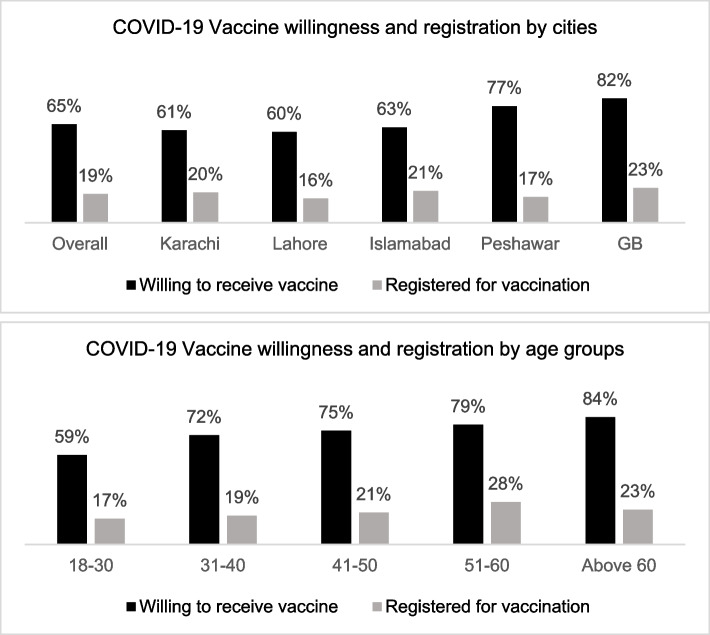
COVID-19 vaccine willingness and registration by city and age

Among 6,648 respondents who participated in the survey (including those found ineligible, Fig. 1), 3,117 (47%) were willing to receive vaccination. Of these, 2,071 (31% of all interviewed) had registered to receive vaccination and 1,647 (25%) had received at least one dose of the vaccine. Among the 2,270 that completed the interview, 1470 (65%) were willing to receive the vaccine, but only 424 (19%) had registered to receive it (Fig. 2).

In multivariate analysis, the odds of willingness to vaccinate increased with age, tertiary education, among those who were currently working, and among those who felt that COVID-19 risk was moderate or high in their community. They also described themselves as being moderately or highly compliant with SOPs. However, this willingness did not directly correlate with registration to receive the vaccine (kappa: 0.136, p < 0.05) and 75% of those that were willing to receive the vaccine had not yet registered to receive it. Those registered to receive vaccination were more likely 51–60 years old, had tertiary education, had previously experienced COVID-19, and described themselves as highly compliant with SOPs. They also reported having seen information about vaccination on the internet/social media or had received a call or SMS text about it (Table 3).

**Table 3:** Logistic regression adjusted odds ratios for vaccine willingness and registration

	**Willingness to Vaccinate**	**COVID-19 Vaccine Registration**
	**aOR (95% CI)**^**a**^	**aOR (95% CI)**^**a**^
**Sex (Male)**		
Female	0.78 (0.58, 1.06)	0.80 (0.54, 1.16)
**Age group (18—30)**		
31—40	1.90* (1.44, 2.51)	1.17 (0.86, 1.60)
41—50	2.84* (1.87, 4.32)	1.46 (0.98, 2.16)
51—60	2.88* (1.52, 5.44)	2.37* (1.38,4.08)
Above 60	6.48* (1.94, 21.58)	1.01 (0.37, 2.74)
**Educational level**^**b**^** (None or less than primary)**		
Primary to middle	0.99 (0.70, 1.40)	0.78 (0.52, 1.18)
Secondary to intermediate	1.10 (0.81, 1.48)	0.87 (0.62, 1.23)
Tertiary	2.02* (1.36, 3.01)	1.75* (1.18, 2.6)
**Occupation (Not working)**		
Working	1.34* (1.01, 1.78)	1.27 (0.90, 1.79)
**Self-employed (Small business)**		
Large businessmen	4.94 (0.62, 39.44)	0.91 (0.23, 3.66)
**Salaried (Unskilled worker)**		
Low skilled worker	0.95 (0.64, 1.38)	1.13 (0.73, 1.75)
Skilled worker	1.32 (0.69, 2.51)	1.45 (0.78, 2.69)
**Socioeconomic status (High)**		
Upper Middle	1.50 (0.92, 2.47)	1.52 (0.88, 2.61)
Middle	1.37 (0.91, 2.06)	1.16 (0.74, 1.81)
Lower Middle	1.41 (0.96, 2.07)	1.13 (0.72, 1.78)
Low	1.49 (0.92, 2.42)	1.47 (0.86, 2.51)
**Source of information**		
TV	0.85 (0.68, 1.05)	0.82 (0.64, 1.06)
Internet/social media	1.17 (0.92, 1.48)	1.3* (1.02, 1.74)
Word of mouth/Family/friends	1.03 (0.81, 1.33)	1.07 (0.80, 1.43)
Health provider	1.45 (0.99, 2.14)	1.59 (0.02, 1.09)
Call/SMS	1.24 (0.77, 1.98)	2.24* (1.44,3.48)
Newspaper	1.89 (0.97, 3.69)	0.62 (0.30, 1.31)
Government institutes	2.32* (1.06, 5.05)	1.83 (0.99, 3.37)
Radio^c^	-	0.98 (0.17, 5.61)
None	0.8 (0.6, 1.2)	0.8 (0.5, 1.2)
**COVID-19 risk perception (Not risky at all)**		
Moderately risky	2.71* (1.50, 4.91)	1.74 (0.80, 3.80)
Yes, extremely	4.38* (2.70, 7.12)	1.76 (0.89, 3.47)
**Prior experience of COVID-19: self or family member (No)**		
Yes	1.55 (0.92, 2.64)	1.95* (1.23, 3.1)
**SOP compliance score (Low compliance)**		
Moderate compliance	1.62* (1.16, 2.27)	1.46 (0.92, 2.31)
High compliance	1.72* (1.26, 2.35)	1.62* (1.05, 2.50)

^*^Values significant at *p* < 0.05. Robust standard errors are used. The categories in brackets after a variable name are base categories. The coefficients show likelihood odds ratios of outcomes in comparison with base categories of every predictor

^a^Multivariate logistic regression adjusted for sex, age group, education, and occupation

^b^Primary to middle: grade < 9; Tertiary: Bachelor or Master

^c^Logistic regressions were omitted because of collinearity

### Reasons for COVID-19 vaccine hesitancy and motivation

Among respondents that either did not intend to receive vaccination (*n* = 663, 29%) or were unsure (*n* = 137, 6%), 36% felt that they did not need vaccination and 31% were concerned about vaccine safety or side effects. Among those who intended to receive the vaccine, 70% did so to protect their own health, while 24% wanted to help curb the pandemic (Table 4).

**Table 4:** Reported COVID-19 vaccine hesitancy and motivation reasons

VARIABLES	Frequency (%)
**Reasons for Hesitancy (** ***N*** ** = 800)**	
No need	284 (35.5%)
Vaccine safety and side effects	251 (31.4%)
Not specific reason	67 (8.4%)
Lack of information	45 (5.6%)
There is no covid	43 (5.4%)
Medical conditions	35 (4.4%)
No permission from home	35 (4.4%)
Religious beliefs	32 (4%)
No time	21 (2.6%)
When majority have vaccinated	10 (1.3%)
**Reasons for Motivation (** ***N*** ** = 1470)**	
For health safety	1029 (70%)
To end the pandemic	357 (24.3%)
Enforced by employer	197 (13.4%)
To protect others	154 (10.5%)
Exemption from following SOPs	59 (4%)
To travel abroad	43 (2.9%)
To go to educational institutes	28 (1.9%)
Religious reasons	9 (0.6%)
No specific reason	158 (10.7%)
As it is free	6 (0.4%)

Willingness to vaccinate was high in Peshawar and GB, increased with age, and among those with high education and the employed (Table 5). It increased among those who felt the COVID-19 was a risk, who followed government-prescribed precautions, and those who had experienced it in a family member (but not necessarily themselves). This willingness however, did not always translate into registrations to receive the vaccine. Only those in the 51–60 age bracket, with a higher degree, and had previously had the infection, had registered for vaccination.

**Table 5:** Multivariate analysis of predictors of willingness and registration to receive COVID-19 vaccination

VARIABLES	Willingness to Vaccinate	Vaccine Registration
**City (Islamabad)**		
Lahore	0.856 (0.623, 1.174)	0.844 (0.591, 1.205)
Peshawar	1.614* (1.109, 2.348)	0.735 (0.490, 1.101)
Karachi	1.08 (0.786, 1.485)	1.097 (0.776, 1.551)
Gilgit Baltistan	2.077* (1.107, 3.898)	1.133 (0.625, 2.055)
**Sex (Male)**		
Female	0.774 (0.554, 1.081)	0.713 (0.473, 1.073)
**Age Group (18—30)**		
31—40	1.915* (1.416, 2.590)	1.138 (0.819, 1.580)
41—50	2.775* (1.729, 4.455)	1.383 (0.910, 2.102)
51—60	3.091* (1.510, 6.329)	2.135* (1.201, 3.795)
Above 60	6.432* (2.206, 18.75)	0.779 (0.273, 2.224)
**Education level (None/less than primary)**		
Primary to middle	0.971 (0.669, 1.410)	0.794 (0.515, 1.223)
Secondary to intermediate	1.146 (0.826, 1.590)	0.877 (0.610, 1.261)
Tertiary (Bachelor/Master)	1.811* (1.184, 2.770)	1.586* (1.031, 2.438)
**Employment status (Unemployed/ student/housewife)**		
Employed/unskilled/low skilled	1.430* (1.037, 1.971)	1.409 (0.966, 2.055)
Self-employed	1.084 (0.758, 1.551)	1.151 (0.759, 1.745)
**COVID-19 risk perception (Not risky at all)**		
Moderately risky	2.443* (1.265, 4.719)	1.562 (0.707, 3.451)
Yes, extremely risky	4.501* (2.607, 7.768)	1.566 (0.790, 3.105)
**Previous self-infection of COVID-19 (No)**		
Yes	1.363 (0.649, 2.863)	2.034* (1.004, 4.117)
**Previous infection of family members (No)**		
Yes	2.249* (1.014, 4.987)	1.537 (0.839, 2.816)
**SOP Score (Low compliance)**		
Moderate compliance	1.433 (0.969, 2.120)	1.542 (0.942, 2.524)
High compliance	1.624* (1.121, 2.353)	1.748* (1.092, 2.798)
** Constant**	0.207* (0.100, 0.429)	0.0767* (0.0307, 0.192)
** Observations**	1,903	2,014

^*^Values significant at *p* < 0.05. Robust standard errors are used. The categories in brackets after a variable name are base categories. The coefficients show likelihood odds ratios of outcomes in comparison with base categories of every predictor

## Discussion

In this nationwide study of major urban cities of Pakistan in June 2021, lived experience of COVID-19 infections was uncommon in that only 3.6% reported being infected and 5% had experienced it in a family member. Willingness to receive the vaccination was 65%, and of these, two-thirds had registered themselves to receive the vaccine. We found that willingness to receive COVID-19 vaccination increased with age, education, and employment, but was similar among both sexes.

In our sample, 80% or more knew about COVID-19 and its vaccine. Television, social media and family/friends were the main sources of information, whereas government, work, or school-related sources were less important, in contrast with the neighboring country India where government officials were primary sources of information rather than social media or family/friends [19]. This might perhaps be reflecting the long periods of lockdowns that have forced people away from work and schools, and towards televisions, mobile phones, or families. Government sources are mistrusted in Pakistan as they are in the region [20, 21]. The popularity of social or conventional mass media is a departure from other public health areas in Pakistan, e.g., family planning, where such approaches have had a more modest impact [22].

Despite awareness, only around half of the respondents said they were willing and two-thirds of those had registered to receive the vaccine. This is lower than seen in other studies from Pakistan that focused on specific respondents [23], USA [24], UK [25] and Europe, China and Indonesia, but higher than in Jordan and Southern Ethiopia [20, 26–29]. In our sample, 1 out of 3 respondents (35%) felt that a vaccine was not needed and slightly fewer (31%) feared either vaccine safety or its side effects. This level of hesitancy is higher than results from USA (11% were hesitant and 32% were unsure) and UK (9% and 27%).

Individuals who had experienced the detriments on COVID-19 were more willing to get vaccinated, consistent with other studies emphasizing that motivation to vaccination is influenced by individual perception based on experience [30–32]. However, only a first-hand experience with the disease, and not of the family, was associated with taking action, i.e., registering to get vaccinated. Similar to our results that individuals who perceived COVID-19 infection as riskier were more willing to get vaccinated, previous studies conducted in Asia report perceived risk to a deadly infection being associated with positive support for vaccinations [31, 32].

Since vaccination intention is context-specific [33] and influenced by COVID-19 burden [9], a lack of “lived experience”, less than 5% reported infection for self or close family members, may have contributed to the overall low acceptance of vaccination. The country was between its 3^rd^ and 4^th^ waves of COVID-19 and there were fewer infections in June 2021 when this survey was conducted. Vaccination rates increased nationwide a month later in July 2020, following a resurgence in cases and hospitalizations [34]. Additionally, at least some of the hesitancy, reasoned by ‘vaccine safety and side effects concerns’ in our study, may have been fueled by recent media coverage of thrombosis associated with the AstraZeneca vaccination in age groups below 40 years [35, 36].

Willingness and registration to receive vaccination increased with age, education, and employment. More severe complications and higher mortality from COVID-19 infection with increasing age and associated chronic diseases have been well-recognized in Pakistan and elsewhere, which leads to increased willingness to vaccinate [25, 37–39]. However, mobility concerns, possible difficulty in registration process, and increased dependency on younger family members might be associated with lesser registration in older adults despite the highest willingness. Tertiary level education was associated with both the highest intentions and registrations for vaccination; anecdotal findings suggested that this group also retained the highest demand for vaccination [40]. Employment may also have driven vaccination through government mandates. In June, just before our data collection period, Government of Pakistan announced it would withhold the salaries of unvaccinated government employees [41] and many private employers also mandated vaccination [42]. Those intending to receive vaccine also showed higher awareness of risks and SOPs compliance. Despite patriarchy manifesting as avoidance of vaccination of women for the fear of causing infertility [43, 44], along with some evidence of lower willingness to vaccinate among women [24, 40, 45–48], we found no difference in vaccination intention across sexes.

### Strengths and limitations

This study was large, included representative samples from major urban centers of Pakistan, and provided timely evidence to policymakers engaged in Pakistan’s COVID-19 response. However, our study has many limitations. The survey was undertaken during a highly dynamic period when vaccinations were being expanded to increasingly lower age groups. Lack of current eligibility of at least some respondents may have influenced their answers. Telephonic surveys, although necessary during global pandemic, excludes the population that either does not possess a phone or resides outside the coverage areas. However, mobile phone usage in Pakistan is more than 80% [49] and higher in urban areas, which were the focus of this study. This limitation was best covered by conducting the survey through Jazz mobile company, that has the largest customer-base in the country.

Our findings might have suffered from non-response bias. During the eligibility process, 20% of all those approached reported not having heard of COVID-19 and another 5% had not heard of the government’s vaccination program. It is likely that at least some of those that claimed to not know of COVID-19 in mid-June 2021, did so just to not continue with the interview, as it is considered culturally inappropriate to refuse directly. In a community face-to-face study in urban Rawalpindi and Islamabad at the same time, only around 3% of respondents had not heard of COVID-19 (manuscript in preparation). Despite the high refusal and ineligibility, our sample corresponded to the socio-demographic distribution of the population, and we ensured weightage during analysis to account for the city-wise population and socioeconomic distribution.

### Policy implications

We suggest several concrete policy implications in Pakistan. Firstly, communication strategy focusing on youth and women, is important to address overall COVID-19 vaccination hesitancy. Messages should be tailored to address concerns regarding vaccine safety, side-effects, myth busting and importance of vaccination in youth. Secondly, to combat possible difficulty in registration and access to centers for older adults, mobile vaccination vans should be exploited, and national volunteer networks should be engaged to assist older adults in vaccination registration. Thirdly, involvement of government institutes is highly necessary in influencing the public. This urges the frequency in releasing updated statistic on the current burden of COVID-19 and the current vaccinated individuals in the country to further motivate others to increase willingness and registration for the COVID-19 vaccine. Lastly, respondents’ reliance on health workers, social media, friends, and families as information sources imply leveraging general pro-vaccination stance to increase COVID-19 vaccination uptake. Engaging local health workers, insisting them to share credible vaccination experiences, asserting celebrities’ endorsement could be effective social signals in driving positive shift of social norms towards greater vaccination acceptance.

## Conclusion

Our findings suggest several policy implications in Pakistan. Since there are no particular myths or misconception-based reservations to vaccination, communication must focus on promoting the value of vaccination rather than to overcome fears. More importantly, given the relatively (with respect to many other countries) low lived experience with the virus in many communities, perhaps a community-based approach, for example, mobile outreach may be applied to low-caseload communities. This may also help vaccinate women who tend to have lower mobility and access to healthcare. The approach would also suggest the need to include community organizations and grass-root workers in mobilizing the vaccination effort. Future research may include a formal evaluation of how effective each of these approaches is in overcoming hesitancy and mobilizing for vaccinations.

## Supplementary Information

**Additional file 1: Table S1. **Survey tool used in the study.

## Data Availability

The dataset analysed during this study is included in this published article (Additional file 1).

## Abbreviation

ARPU: Average Revenue Per User
