# Supplementary material for: COVID-19 vaccine hesitancy and attitudes in Pakistan: a cross-sectional phone survey of major urban cities
Source: BMC Public Health. 2023 Jun 9;23:1112. doi: 10.1186/s12889-023-15905-3 (PMC10252162; doi:10.1186/s12889-023-15905-3)
Supplement: Supplementary file 1 — Additional file 1: Table S1. Survey tool used in the study. [file 12889_2023_15905_MOESM1_ESM.docx]

**Table S1. Survey tool used in the study**

| **Category** | **Serial no.** | **Question** | | | **Response** |
| --- | --- | --- | --- | --- | --- |
| **Socio - demographics** | **1** | Name (optional) | | |  |
|  | **2** | Age | | |  |
|  | **3** | Gender | | |  |
|  | **4** | Home Address | | |  |
|  | **5** | Highest level of Education | | | None Primary (6 years or less) Secondary (7 to 10 years) Higher Secondary (10 to 12 years) Bachelor (BA, BSc, B.Com, B.ED - 13 to 14 years)  Master (MA, MSc, M.ED - 15 to 16 years) MPhil, MS (17 to 18 years) Doctorate (> 18 years) |
|  | **6** | City | | | Karachi  Lahore  Islamabad  Peshawar  Gilgit |
|  | **7** | Employment status | | | Unemployed  Student  Retired  Housewife  Small business  Large business  Unskilled worked  Low skilled worker  Skilled worker |
| **Disease Awareness and SOP Compliance** | **8.1** | Have you ever been affected by COVID-19? | | | Yes  No Do not know |
|  | **8.2** | Has someone in your family ever been affected by COVID-19? | | | Yes  No Do not know |
|  | **8.3** | Ask if 8.1 or 8.2 = Yes | | How severe was the infection? | Hospitalized  Not hospitalized  Do not know |
|  | **9** | Do you agree with the following statement: I am worried that I will contract COVID-19. | | | Strongly Agree Agree Undecided Disagree Strongly Disagree |
|  | **10** | Do you follow social distancing precautions (6-feet distancing, open air meet-ups, outdoor dining) | | | Always Often Sometimes Rarely Never |
|  | **11** | Do you wear a mask when you leave your house? | | | Always Often Sometimes Rarely Never |
|  | **12** | Do you work from home? | | | a) Yes Sometimes  Never  b) If sometimes, state number of days per month |
|  | **13** | Does your workplace work at full capacity? | | | Yes  Somewhat No Do not know |
|  | **14** | Does your workplace follow stringent SOPs? | | | Yes  To some extent No Do not know |
| **Source of information** | **15** | What is your primary source of information for COVID-19? | | | Television Radio Social Media or websites SMS and Calls Newspapers Family and/or community members Doctors, medical experts or community health workers Religious leaders Government officials Other (specify) |
|  | **16** | Which of these information sources do you trust most? | | | Television Radio Social Media or websites SMS and Calls Newspapers Family and/or community members Doctors, medical experts or community health workers Research papers/ journal articles Religious leaders Government officials/government sources Other (specify) |
| **Vaccine Awareness and Receptivity** | **17** | Do you know that vaccination for COVID-19 in Pakistan has begun? | | | Yes  No Not sure |
|  | **18** | Are you aware of more than one COVID-19 vaccine? | | | Yes  No Not sure |
|  | **19** | Which of the following COVID-19 vaccines do you know about? | | | Pfizer Moderna Sinopharm CanSino Sputnik Sinovac AstraZeneca Don´t know the names All of the above |
|  | **20** | Which one of the following vaccines do you think are safe for use? | | | China Manufactured Europe/US manufactured  UK manufactured   Russia-Manufactured  Others (specify)  None All of the above |
|  | **21** | Are you willing to get your family vaccinated against COVID-19? | | | Yes  No Other |
|  | **22** | Would you be willing to take COVID-19 vaccine offered by the government? | | | Yes No  Other |
|  | **22a.i** | Ask if 22=’Yes’ | How would you describe your willingness to get vaccinated? | | As soon as possible Wait and see Only if required |
|  | **22a.ii** |  | What are your motivation to get vaccinated? | | Travel (for leisure/for work)  Daily mobility To improve your health To end pandmic Lesser fear of death Freedom to live without following SOPs Social life  Religious tourism (Hajj, umrah, shrines etc) Religious congregational events (friday prayers etc) Freedom to attend educational institutes Other (please specify) |
|  | **22a.iii** |  | Out of all the vaccines that you are aware of, which one would you prefer? | | China Manufactured Europe/US manufactured  UK manufactured   Russia-Manufactured  Government recommended No particular preference Others (specify) |
|  | **22a.iv** |  | Will you continue following SOPs after getting vaccinated? | | Yes  To some extent No Do not know |
|  | **22a.v** |  | Do you think information on COVID-19 (disease + vaccine) is readily available? | | Yes  To some extent No Do not know |
|  | **22a.vi** |  | (Are you aware of) Do you have a nearby vaccination facility | | Yes  No Do not know |
|  | **22b.i** | Ask if 22=’No' | Why? | | Not sure of safety Not sure of efficacy/effectiveness The chance of contracting the virus remains unaltered Fear of side effects such as fever, pain Fear of long term negative health effects  Religious belief  Other (specify) |
|  | **22b.ii** |  | In your opinion, what other reasons might hinder you in getting vaccinated against Covid-19? | | I have no or limited knowledge of the vaccine registration system  The vaccine registration system is difficult to use  There is no vaccination center nearby I do not know where a vaccination center is  It might be too expensive  It will not be available anytime soon  I will not get the brand of vaccine I prefer Other (specify) None applies to me |
|  | **22b.iii** |  | Why do you consider the vaccine to be unsafe? | | Descriptive Answer |
|  | **22b.iv** |  | Is this because of the unavailibility of a vaccine of choice? | | Yes  No  Do not know |
|  | **22b.v** |  | Would you consider getting vaccinated if someone you trust suggested it? | | Yes No Do not know |
|  | **22b.vi** |  | On whose suggestion might you get vaccinated? | | Religious Leaders Politicians Local Doctor Friends  Family Teachers Ministry of Health Provincial Health Department Other (specify)  None |
|  | **22b.vii** |  | How would you like to get more information about COVID-19 vaccine? | | Social media such as WhatsApp  Facebook Instagram Twitter Through telecommunication such as SMS and call  Online platforms such as Zoom, Skype,  Print and Electronic media:TV, newspaper  Face to face communication  Other(specify) |
| **Vaccine registration** | **23** | Have you registered for COVID-19 vaccination? | | | Yes  No Do not know |
